# PTPIP51 crosslinks the NFκB signaling and the MAPK pathway in SKBR3 cells

**DOI:** 10.2144/fsoa-2019-0136

**Published:** 2020-03-04

**Authors:** Eric Dietel, Alexander Brobeil, Claudia Tag, Stefan Gattenloehner, Monika Wimmer

**Affiliations:** 1 Institute of Anatomy & Cell Biology, Justus-Liebig-University, Giessen 35392, Germany; 2 Institute of Pathology, Justus-Liebig-University, Giessen 35392, Germany; 3 Institute of Anatomy, Johannes-Kepler-University Linz, Linz 4040, Austria

**Keywords:** breast cancer, Her2, IKK-16, NFκB, PDTC, protein–protein interactions, PTPIP51

## Abstract

**Aim:**

PTPIP51 interacts with NFκB signaling at the RelA and IκB level. NFκB signaling is linked to the initiation, progression and metastasis of breast cancer. Her2-amplified breast cancer cells frequently display activation of the NFκB signaling. We aimed to clarify the effects of NFκB inhibition on the NFκB- and MAPK-related interactome of PTPIP51 and cell viability in HaCat cells and SKBR3 cells.

**Results:**

IKK-16 selectively reduced cell viability in SKBR3 cells. PDTC induced a formation of the Raf1/14-3-3/PTPIP51 complex in SKBR3 cells, indicating a shift of PTPIP51 into MAPK signaling.

**Conclusion:**

IKK-16 selectively inhibits cell viability of SKBR3 cells. In addition, PTPIP51 might serve as the mediator between NFκB signaling and the MAPK pathway in SKBR3.

## Lay abstract

Breast cancer is the most common cancerous disease among women. Prognosis and therapy of breast cancer depends on the expression of hormone and surface receptors such as Her2, which promote tumor growth and invasion via activation of downstream signaling pathways. NFκB signaling represents a downstream signaling pathway that can be activated by Her2. In this study, we demonstrated that inhibition of NFκB signaling with IKK-16 reduces cell viability in breast cancer cells with amplified Her2. Furthermore, we identified PTPIP51 as a potential mediator of crosstalks between the MAPK pathway and NFκB signaling. This signaling pathway could therefore be a target for future drug development.

The body of evidence stating the importance of NFκB signaling in the initiation, progression and metastasis of several tumor entities is steadily growing [1–4]. Alterations in NFκB signaling can be the consequence of direct mutations of signaling molecules belonging to the NFκB signaling cascade, stimulation of signaling via the inflammatory tumor microenvironment or crosstalk between NFκB signaling and other dysregulated signaling pathways [5–8].

The amplification and overactivation of the Her2 receptor in breast cancer represents a perfect example of the activation of NFκB signaling via the crosstalk of different signaling pathways [8]. About 20–30% of all breast cancers exhibit amplification of the Her2 receptor, accompanied by more aggressive tumor growth and reduced overall survival [9,10]. The Her2 receptor mainly activates two signaling pathways: the MAPK pathway and Akt signaling [9]. Besides these two pathways, Her2 is also capable of activating IKKs [8]. IKKs are essential for the activation of the NFκB signaling cascade via phosphorylation of IκB. Phosphorylation tags IκB for ubiquitinylation and thus triggers its degradation. After the degradation of IκB, the nuclear localization signal of RelA is exposed. Consequently, RelA can exert its transcriptional activity [11,12]. This Her2-induced NFκB activation contributes to the growth of the tumor, the development of therapy resistance and the epithelial–mesenchymal transition, which represents a hallmark in the formation of metastasis [4,8].

It is noteworthy that the scaffold protein, protein tyrosine phosphatase interacting protein 51 (PTPIP51), interacts with both signaling structures – the Her2 receptor and NFκB signaling [13,14]. The interaction of PTPIP51 with the Her2 receptor seems crucial for the sensitivity of Her2-amplified breast cancer cell lines to EGFR/Her2-targeted therapies [14]. Besides the direct interaction with the Her2 receptor, PTPIP51 is involved in the titration of the MAPK signaling [15–17]. Within this pathway, PTPIP51 exerts an activating effect via the binding of Raf1 and 14-3-3 [16]. The formation of the PTPIP51/14-3-3/Raf1 complex induces an activation of ERK1/2, thus an activation of MAPK signaling [15]. The formation of the Raf1/14-3-3/PTPIP51 complex is strictly regulated by the phosphorylation of PTPIP51. Phosphorylation of tyrosine 176 leads to a dissolution of the complex and an omission of the MAPK pathway-stimulating effect. In contrast, the phosphorylation of serine 212 enhances the formation of the ternary complex [15,17,18]. Both phosphorylation sites are under the control of several kinases, including receptor tyrosine kinases (e.g., the EGFR) and nonreceptor kinases (e.g., c-Src) and phosphatases [15,17,18].

The regulation of PTPIP51 in NFκB signaling contradicts the observations made in the MAPK pathway. Here, the formation of the RelA/IκB/PTPIP51 complex inhibits the NFκB signaling [13]. Due to the recency of our knowledge of PTPIP51 function in NFκB signaling, the critical phosphorylation sites, which regulate the binding of PTPIP51 with RelA and IκB, are unknown. Brobei and coworkers showed that stimulation of HaCat cells with TNFα induces a disintegration of the PTPIP51/IκB/RelA complex. Vice versa, inhibition of NFκB signaling led to a formation of the PTPIP51/IκB/RelA complex [13].

Based on these findings, this study aimed to elucidate the interaction shifts of PTPIP51 upon NFκB inhibition in NFκB signaling and their effects on the MAPK pathway using the Duolink proximity ligation assay. NFκB signaling inhibition was performed using pyrrolidine dithiocarbamate (PDTC) and IKK-16, respectively. PDTC was thought to act as an antioxidant and thereby inhibit TNFα-induced NFκB activation. Hayakawa and coworkers showed that PDTC could inhibit ubiquitin ligase activity in a cell-free system, which lacks reactive oxygen species [19]. Thus, the antioxidative properties of PDTC are not needed for the inhibition of NFκB signaling [19,20]. IKK-16 acts as a small molecule inhibitor of IKK1, IKK2 and the IKK complex [21]. Through the inhibition of these serine/threonine kinases, the phosphorylation of IκB is not possible [12] Subsequently, IκB cannot be degraded and RelA cannot exert its transcriptional activity [12]. The impact of the applied agents on cell survival was analyzed by MTT assays. Thus, we were able to describe differential regulations in the Her2-amplified breast cancer cell line SKBR3 and the nontumor keratinocyte cell line HaCat.

## Materials & methods

### Cell culture

SKBR3 cells were purchased from Cell Line Service (Eppelheim, Germany). The cells were cultured in Dulbecco’s MEM (Biochrom, Berlin, Germany) containing 10% fetal calf serum and 1% penicillin/streptomycin in a humidified chamber at 37°C and 5% CO_2_. The medium renewal was performed every 2–3 days. Cell harvesting was performed at a confluence of 70–80% with Accutase. The SKBR3 cells were seeded in culture slides (30,000 cells per well; Falcon CultureSlides, Corning Life Science, NY, USA, Cat.# 354108) or used as indicated for other experiments.

The HaCaT cells were obtained and handled as described in previous publications of our group [13]. Cells were harvested with Trypsin in a humidified chamber at 37°C and 5% CO_2_. Subsequently, the cells were seeded on culture slides (Falcon CultureSlides, Corning Life Science, Cat.# 354108) or 96-well plates (Sigma-Aldrich Chemie GmbH, Taufkirchen, Germany, Cat.# CLS3340).

### Treatment

The cells were allowed to grow for 24 h after seeding. Subsequently, they were treated with different concentrations of ammonium pyrrolidine dithiocarbamate (PDTC) (Sigma-Aldrich, Cat.# P 8765, Munich, Germany) or IKK-16 (Cat.# S2882, Selleckchem, Munich, Germany) (diluted in culture medium) for either 6 or 24 h. The reaction was stopped by removal of medium and addition of ice-cold phosphate-buffered saline. The fixation was performed with ice-cold methanol for proximity ligation assays. The procedure for the MTT assays is described in the MTT subsection.

### Antibodies

All antibodies used are listed in Supplementary Table 1.

### Duolink proximity ligation assay

For evaluation of the interactions of proteins, the Olink Duolink proximity ligation assay (PLA probe anti-rabbit minus, Cat.# DUO92005, PLA probe anti-mouse plus, Cat.# DUO92001, anti-goat plus Cat.# DUO92003, Detection Kit Orange, Cat.# DUO92007, Sigma-Aldrich Chemie GmbH) was used. The assay was carried out according to the manufacturer manual. Leuchowius and coworkers identified the Duolink proximity ligation assay as an adequate tool for the identification of small-molecule effectors for protein–protein interactions [22].

### Fluorescence microscopy

The photo documentation was performed with an Axioplan 2 fluorescence microscope equipped with Plan-Apochromat objectives (Carl Zeiss Jena, Jena, Germany).

### Protein interaction analysis

Quantification was carried out using the DuoLink Image Tool (Olink Bioscience, Uppsala, Sweden, v1.0.1.2). The software identifies Dapi-positive nuclei and counts fluorescence dots in a user-defined cell diameter preset. For each indicated concentration, at least 100 single cells were analyzed in three independent experiments.

### MTT assay

HaCat and SKBR3 cells were seeded at a density of 10,000 cells per well in a 96-well plate. The cells were allowed to grow for 24 h. Cells were treated as indicated. MTT solution was added 4 h before the end of the incubation time. Formazan crystals were solubilized using a solubilization solution (10% SDS in 0.01M HCl). The solution of the crystals was performed overnight in a humidified chamber at 37°C and 5% CO_2_. Assays were evaluated with the Berthold Tech TriStar ELISA Reader (Bad Wildbad, Germany). The assays were performed in quintuplicates.

### Statistical analysis

Data were evaluated using GraphPad Prism 6 software. Statistical significance was determined using ANOVA, followed by Dunnett’s multiple comparison tests. Results were considered significant with p < 0.05. (*[p < 0.05]; **[p < 0.01]; ***[p < 0.001]; ****[p < 0.0001]).

## Results

All experiments were performed with the SKBR3 cell line, an Her2-amplified breast cancer cell line and the spontaneously immortalized keratinocyte HaCat cell line. This setting allows comparison of the effects of NFκB inhibition on the malignantly transformed signaling system in the SKBR3 cell line with the normal signaling in the HaCat cell line.

### Inhibition of NFκB signaling with PDTC or IKK-16 leads to differential regulations of the cell viability in SKBR3 cells & HaCat cells

The effects of NFκB inhibition by the application of PDTC and IKK-16, respectively, were monitored using the MTT assay, which measures cell viability through the formation of formazan crystals. The results were equalized/related to the control value equaling 1. Each experiment was performed using the same dilution of DMSO to exclude the cytotoxic effects of DMSO.

**Figure 1.**
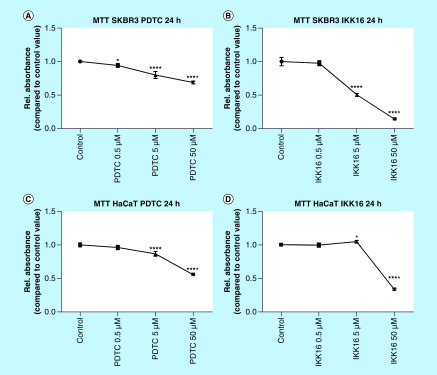
Cell viability of SKBR3 cells and HaCat cells treated with PDTC in concentrations of 0.5, 5 and 50 μM and with IKK-16 in concentrations of 0.5, 5 and 50 μM. **(A)** SKBR3 cells treated with the indicated concentrations of PDTC for 24 h. **(B)** SKBR3 cells treated with the indicated concentrations of IKK-16 for 24 h. **(C)** HaCat cells treated with the indicated concentrations of PDTC for 24 h. **(D)** HaCat cells treated with the indicated concentrations of IKK-16 for 24 h. The graphs show the mean value and standard deviation. *p < 0.05; ****p < 0.0001.

Applying PDTC in increasing concentrations to SKBR3 cells resulted in a significant decrease of the cell viability (0.5 μM p < 0.05; 5 μM p < 0.0001; 50 μM p < 0.0001). Comparable results were seen for the inhibition of the NFκB signaling using PDTC in the HaCat cell line (5 μM p < 0.0001; 50 μM p < 0.0001). The application of IKK-16 induced differing results. The treatment of SKBR3 cells with rising concentrations of IKK-16 resulted in a highly significant decrease in cell viability (5 μM p < 0.0001; 50 μM p < 0.0001). In contrast, applying IKK-16 to HaCat cells led to a slight but significant increase in cell viability for the application of 5 μM (p < 0.05). Increasing the concentration resulted in a highly significant decrease in cell viability (p < 0.0001; Figure 1).

### NFκB inhibition in SKBR3 cells & HaCat cells induced interaction shifts of the RelA/IκB/PTPIP51 complex

The formation of the RelA/IκB/PTPIP51 complex is essential for the titration of the NFκB signaling [13]. To monitor the interaction shifts of the RelA/IκB/PTPIP51 interactome, Duolink proximity ligation assays were performed. Interestingly, the regulation of the RelA/PTPIP51 interaction varied relative to the applied NFκB inhibitor and the used cell line. Of note, the application of 50 μM IKK-16 to the SKBR3 cell line severely diminished the seeded cell population leaving only cell debris. Thus, an adequate evaluation of the Duolink proximity ligation assays for this setting was not possible.

**Figure 2.**
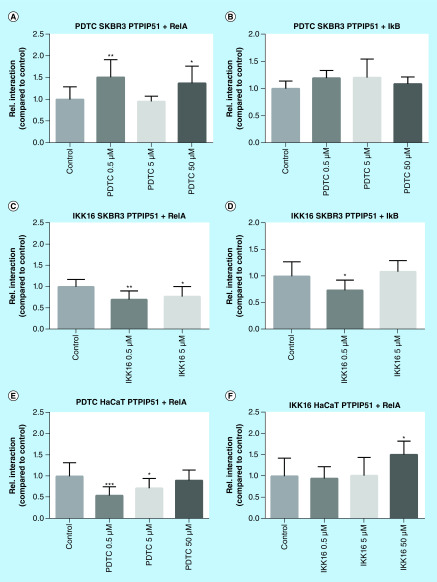
NFκB-related interactome of PTPIP51 in SKBR3 cells and HaCat treated with PDTC in concentrations of 0.5, 5 and 50 μM and with IKK-16 in concentrations of 0.5 and 5 μM. **(A)** Interaction of PTPIP51 and RelA in SKBR3 cells treated with PDTC in the indicated concentrations for 6 h. **(B)** Interaction of PTPIP51 and IκB in SKBR3 cells treated with PDTC in the indicated concentrations for 6 h. **(C)** Interaction of PTPIP51 and RelA in SKBR3 cells treated with IKK-16 in the indicated concentrations for 6 h. **(D)** Interaction of PTPIP51 and IκB in SKBR3 cells treated with IKK-16 in the indicated concentrations for 6 h. **(E)** Interaction of PTPIP51 and RelA in HaCat cells treated with PDTC in the indicated concentrations for 6 h. **(F)** Interaction of PTPIP51 and RelA in HaCat cells treated with IKK-16 in concentrations of 0.5, 5 and 50 μM for 6 h. The graphs show the mean value and standard deviation. *p < 0.05; **p < 0.01; ***p < 0.001.

Application of PDTC to SKBR3 cells induced a significant increase of the RelA/PTPIP51 interaction for the lowest and highest concentration used in this study (0.5 μM p < 0.01; 50 μM p < 0.05). In contrast, HaCat cells submitted to the same agent displayed a highly significant reduction in RelA/PTPIP51 interactions (0.5 μM p < 0.001; p < 0.05). All applied concentrations of IKK-16 significantly reduced the RelA/PTPIP51 interaction (0.5 μM p < 0.01; 5 μM p < 0.05) in the breast cancer cell line SKBR3. On the contrary, the application of IKK-16 to HaCat cells enhanced the interaction of RelA and PTPIP51 for the highest tested concentration (50 μM p < 0.05). The regulation of the RelA/IκB/PTPIP51 complex was further evaluated by monitoring the interaction of PTPIP51 and IκB in SKBR3 cells. None of the tested PDTC concentrations affected the interaction of PTPIP51 and IκB. In contrast, treatment with IKK-16 led to a significant reduction of PTPIP51/IκB interaction (0.5 μM p < 0.05; Figure 2).

### Selective IKK inhibition by IKK-16 enhances the interaction of PTPIP51 & the Her2 receptor

**Figure 3.**
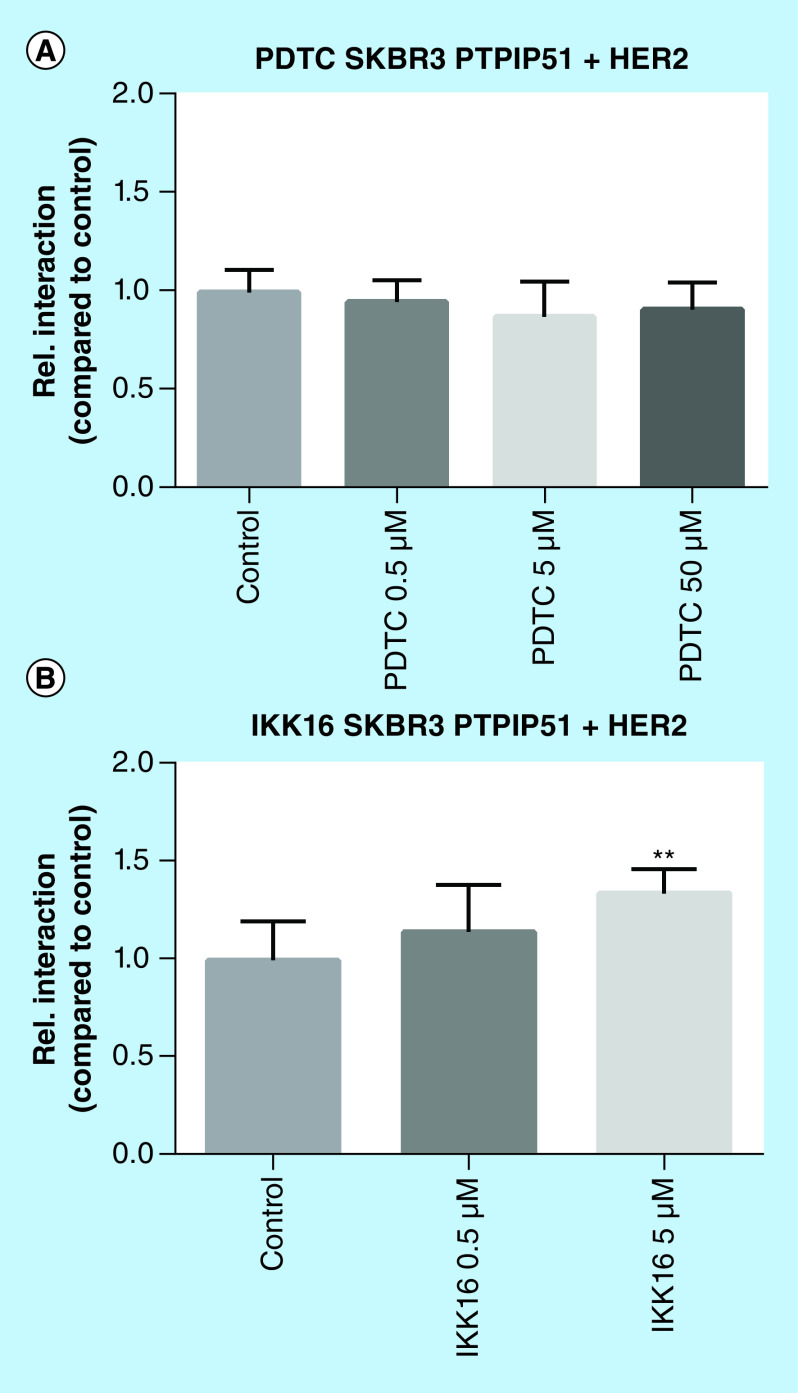
Interaction of PTPIP51 and the Her2 receptor in SKBR3 cells treated with PDTC (0.5, 5 and 50 μM) and IKK-16 (0.5 and 5 μM). **(A)** Interaction of PTPIP51 and the Her2 receptor in SKBR3 cells treated with PDTC in the indicated concentrations for 6 h. **(B)** Interaction of PTPIP51 and the Her2 receptor in SKBR3 cells treated with IKK-16 in the indicated concentrations for 6 h. The graphs show the mean value and standard deviation. **p < 0.01.

The amplified Her2 receptor activates the NFκB signaling via the canonical pathway and the activation of IKKα [8]. PTPIP51 interacts with the Her2 receptor and seems to be crucial for the responsiveness of Her2 amplified breast cancer cells toward Her2 targeted therapies [14]. Thus, we examined the interaction of PTPIP51 and the Her2 receptor under NFκB inhibition. The application of PDTC to SKBR3 cells for 6 h did not affect the interaction of PTPIP51 and Her2. In contrast, the IKK-16 treatment of SKBR3 cells significantly enhanced the interaction of PTPIP51 and the Her2 receptor (5 μM p < 0.01; Figure 3).

### Inhibition of NFκB signaling induced interaction shifts in the MAPK-related PTPIP51 interactome

**Figure 4.**
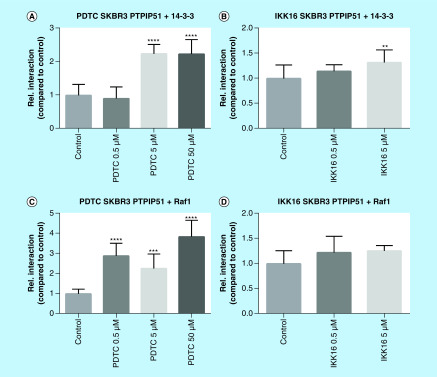
MAPK-related interactome of PTPIP51 in SKBR3 cells treated with PDTC (0.5, 5 and 50 μM) and IKK-16 (0.5 and 5 μM). **(A)** Interaction of PTPIP51 and 14-3-3 in SKBR3 cells treated with PDTC in the indicated concentrations for 6 h. **(B)** Interaction of PTPIP51 and 14-3-3 in SKBR3 cells treated with IKK-16 in the indicated concentrations for 6 h. **(C)** Interaction of PTPIP51 and Raf1 in SKBR3 cells treated with PDTC in the indicated concentrations for 6 h. **(D)** Interaction of PTPIP51 and Raf1 in SKBR3 cells treated with IKK-16 in the indicated concentrations for 6 h. The graphs show the mean value and standard deviation. **p < 0.01; ***p < 0.001; ****p < 0.0001.

**Figure 5.**
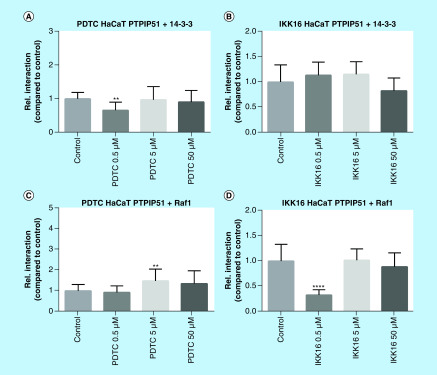
MAPK-related interactome of PTPIP51 in HaCat cells treated with PDTC (0.5, 5 and 50 μM) and IKK-16 (0.5 and 5 μM). **(A)** Interaction of PTPIP51 and 14-3-3 in HaCat cells treated with PDTC in the indicated concentrations for 6 h. **(B)** Interaction of PTPIP51 and 14-3-3 in HaCat cells treated with IKK-16 in the indicated concentrations for 6 h. **(C)** Interaction of PTPIP51 and Raf1 in HaCat cells treated with PDTC in the indicated concentrations for 6 h. **(D)** Interaction of PTPIP51 and Raf1 in HaCat cells treated with IKK-16 in the indicated concentrations for 6 h. The graphs show the mean value and standard deviation. **p < 0.01; ****p < 0.0001.

Besides the activation of NFκB signaling, activation of the Her2 receptor is mainly channeled to the activation of the MAPK pathway, especially ERK signaling [9]. Furthermore, Brobeil and coworkers identified PTPIP51 as a crosslink between the NFκB signaling and the MAPK pathway [13]. Therefore, we examined the influence of NFκB inhibition on the MAPK-related interactome of PTPIP51. Application of PDTC to SKBR3 cells resulted in a highly significant increase of PTPIP51/14-3-3 and PTPIP51/Raf1 interaction (PTPIP51/14-3-3 5 μM p < 0.0001; 50 μM p < 0.0001; PTPIP51/Raf1 0.5 μM p < 0.0001; 5 μM p < 0.001; 50 μM p < 0.0001). Of note, inhibition of NFκB signaling using IKK-16 did not affect the interaction of PTPIP51 and Raf1. The PTPIP51/14-3-3 interaction was augmented for the highest applied IKK-16 concentration (5 μM p < 0.01). Inhibition of the NFκB signaling in HaCat cells did not severely affect the MAPK-related interactome of PTPIP51. Application of PDTC to HaCat cells reduced the PTPIP51/14-3-3 interaction for the lowest applied concentration (0.5 μM p < 0.01) and enhanced the PTPIP51/Raf1 interaction if 5 μM PDTC were applied (p < 0.01). Treating HaCat cells with IKK-16 only affected the PTPIP51/Raf1 interaction for the lowest applied concentration (0.5 μM p < 0.0001; Figures 4 & 5).

### Sensitivity toward IKK-16-induced NFκB inhibition correlates with the interaction changes of PTPIP51 & its crucial phosphatase PTP1B

**Figure 6.**
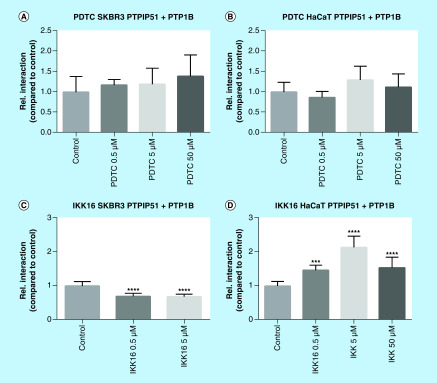
Interaction of PTPIP51 and PTP1B in SKBR3 cells and HaCat cells treated with PDTC (0.5, 5 and 50 μM) and IKK-16 (0.5, 5 and 5 μM). **(A)** Interaction of PTPIP51 and PTP1B in SKBR3 cells treated with PDTC in the indicated concentrations for 6 h. **(B)** Interaction of PTPIP51 and PTP1B in HaCat cells treated with PDTC in the indicated concentrations for 6 h. **(C)** Interaction of PTPIP51 and PTP1B in SKBR3 cells treated with IKK-16 in the indicated concentrations for 6 h. **(D)** Interaction of PTPIP51 and PTP1B in HaCat cells treated with IKK-16 in the indicated concentrations for 6 h. The graphs show the mean value and standard deviation. ***p < 0.001; ****p < 0.0001.

To exert its scaffold protein properties, the phosphorylation of PTPIP51 is tightly regulated by several kinases and phosphatases. For the interaction of PTPIP51 with 14-3-3 and Raf1, the tyrosine residue 176 and the serine residue 212 are needed. While the phosphorylation of Tyr176 prevents the interaction of PTPIP51 and Raf1, the phosphorylation of Ser212 augments the interaction. The critical phosphorylation sites for the interaction with RelA and IκB, respectively, are up to now not known. Since both the NFκB- and the MAPK-related interactome are affected by the inhibition of NFκB signaling, we examined the interaction of PTPIP51 and its crucial phosphatase PTP1B in SKBR3 and HaCat cells. The application of PDTC to SKBR3 and HaCat cells did not affect the interaction of PTPIP51 and PTP1B. Interestingly, the IKK-16 treatment of SKBR3 and HaCat cells led to divergent results. While, IKK-16 inhibited the interaction of PTPIP51 and PTP1B in SKBR3 cells (0.5 μM p < 0.0001; 5 μM p < 0.0001), in HaCat cells the interaction was augmented (0.5 μM p < 0.001; 5 μM p < 0.0001; 50 μM p < 0.0001) (Figure 6).

## Discussion

The role in tumor initiation, progression and the formation of metastasis make NFκB signaling a new target for novel therapeutic agents. In this study, we emphasize the importance of choosing the right agent for targeting the NFκB signaling in Her2-amplified breast cancer cells.

The selective inhibition of IKKs using the small molecule inhibitor IKK-16 induced severe impairments in the cell viability of the Her2-amplified breast cancer cell line SKBR3. Of note, the application of 5 μM IKK-16 led to diametrically opposite results in the investigated cell lines. While the HaCat cells displayed an increase in cell viability, the cell viability of SKBR3 cells was highly significantly reduced. This disparity was further analyzed by the evaluation of the RelA/IκB/PTPIP51 complex using the Duolink proximity ligation assay. Here, the results differed not only between the cell lines but also between the applied agent. Brobeil and coworkers stated the stimulation of the NFκB signaling via application of TNFα results in a dissolution of the RelA/IκB/PTPIP51 complex in HaCat cells, indicating an activation of the NFκB signaling [13]. The inhibition of NFκB signaling via IKK-16 in HaCat cells led to results corroborating the theory of a RelA/IκB/PTPIP51 complex formation and disintegration under inhibition and stimulation of NFκB signaling. Comparable observations were made for the application of PDTC to SKBR3 cells. The regulation under NFκB inhibition using IKK-16 in SKBR3 cells entirely opposes the known regulations of PTPIP51 in NFκB signaling. Here, the interaction of PTPIP51 with RelA and IκB, respectively, is reduced, implying an activation of the NFκB signaling. These observations potentially depict an overshooting counter-regulation against the inhibition of IKKs. Up to now, the mechanisms of these regulations are unknown. The evaluation of the NFκB-related interactome of PTPIP51 is not sufficient to explain the different effects on the cell viability by the applied agents in the two cell lines.

The MAPK pathway is one of the essential growth and proliferation promoting pathways in Her2-amplified breast cancer cells [9]. PTPIP51 plays a pivotal role in the titration of the MAPK pathway activation [15–18]. The regulation of the MAPK-related PTPIP51 interactome upon NFκB inhibition significantly differs between the two cell lines. The application of PDTC to SKBR3 cells shifted PTPIP51 into the Raf1/14-3-3/PTPIP51 complex, indicating the activation of MAPK signaling. This shift was not observed under IKK-16 treatment. Thereby, the non-activation of MAPK signaling explains the severe impairment of cell viability in SKBR3 cells under IKK-16 treatment. In the HaCat cell line, neither of the applied agents led to a remarkable shift of PTPIP51 into the MAPK pathway. These findings depict a potential evasion mechanism of SKBR3 cells against the PDTC mediated NFκB inhibition.

For the precise understanding of these regulations, the exact targets of the applied agents have to be identified. As mentioned in the introduction, PDTC exhibits a NFκB inhibitory property besides its antioxidative effect. IKK-16 is a small molecule inhibitor of the IKK-1, IKK-2 and IKK complex. Through the inhibition of these kinases, phosphorylation of IκB cannot be performed. Subsequently, IκB cannot be degraded and RelA cannot exert its transcriptional activity [12]. Of note, IKK2 is capable of phosphorylating both IκB and PTPIP51. The group-based prediction system (GPS 3.0; http://gps.biocuckoo.org/; [23]) revealed that IKK2 could phosphorylate PTPIP51 at serine 212, which enhances the interaction of PTPIP51 with MAPK signaling on the Raf1 level.

The application of PDTC to the Her2-amplified breast cancer cell line SKBR3 induces inhibition of the IκB ubiquitin ligase resulting in an enhanced interaction of PTPIP51 and RelA. Since IKK activity is not inhibited, but in contrast is even enhanced in the Her2-overactivated setting, IKK2 is still capable of phosphorylating the serine 212 of PTPIP51. The phosphorylation of PTPIP51 at serine 212 forces PTPIP51 into the Raf1/14-3-3/PTPIP51 complex and subsequently leads to a stimulation of MAPK signaling [15,17,18]. Thereby, SKBR3 cells potentially evade the NFκB inhibition via the crosstalk with the MAPK signaling mediated by PTPIP51.

The NFκB inhibition via IKK-16 blocks the phosphorylation of serine 212 of PTPIP51 through IKK2 and thereby the translocation of PTPIP51 into the MAPK signaling. These regulations explain the severe reduction in SKBR3 cell viability under IKK-16 treatment since the blocked NFκB signaling cannot be bypassed by PTPIP51-induced MAPK stimulation.

The interaction of PTPIP51 with Raf1 and 14-3-3 is not only subjected to the serine 212 phosphorylation of PTPIP51 but also to the tyrosine 176 phosphorylation of PTPIP51 [15,17,18]. A crucial regulator of this phosphorylation site is the PTP1B [15,17,18,24]. Interestingly, the interaction of PTPIP51 and PTP1B depends on the level of NFκB inhibition. The inhibition of IKKs leads to different regulations of the PTPIP51/PTP1B interaction in SKBR3 cells and HaCat cells, respectively. This perfectly correlates with the effects on cell viability. The functional implications of these interaction shifts remain unclear since the observed reduction of PTPIP51/PTP1B interaction in SKBR3 cells implies a reduced interaction with the MAPK pathway due to the enhanced phosphorylation of tyrosine 176 of PTPIP51. In contrast, the interaction of PTPIP51 and 14-3-3 was even enhanced under IKK inhibition. The precise mechanisms of this regulation and the effects on phosphorylation of PTPIP51 need further investigation.

**Figure 7.**
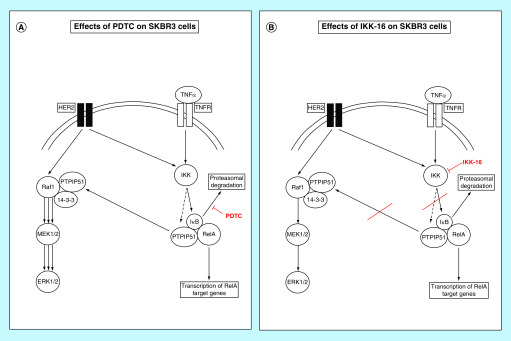
Schematic overview of the interaction shifts in SKBR3 cells after the application of PDTC and IKK-16. **(A)** Application of PDTC to SKBR3 cells inhibits the IκB ubiquitin ligase, thus inhibiting the degradation of IκB. The activation of IKKs through the Her2 receptor potentially leads to phosphorylation of PTPIP51 at serine 212. This mechanism shifts PTPIP51 into MAPK signaling and induces the formation of the Raf1/14-3-3/PTPIP51 complex. The PTPIP51 induced activation of the MAPK pathway bypasses the blocked NFκB signaling. **(B)** Selective inhibition of IKK1, IKK2 and the IKK complex with IKK-16 inhibits the phosphorylation of IκB. Likewise, IKK is not able to phosphorylate PTPIP51 at serine 212 and, thus, PTPIP51 is not shifted into MAPK signaling. Due to the blockage of the aforementioned bypass mechanism, SKBR3 cell viability is severely reduced by IKK-16.

In Her2-amplified breast cancer cells, the activation of IKKs is tightly linked to the overactivation of the Her2 receptor [8]. Recent studies of our group substantiated an interaction of PTPIP51 and the Her2 receptor. Interestingly, selective inhibition of the Her2 receptor using Mubritinib induced a formation of a ternary complex consisting of PTPIP51, c-Src and Her2, which potentially represents a resistance mechanism against Her2-targeted tyrosine kinase inhibitors [14]. The inhibition of IKKs induced a similar enhanced interaction of Her2 and PTPIP51, whereas the PDTC mediated IκB ubiquitin ligase inhibition left the PTPIP51/Her2 interaction unaffected. A schematic overview of the mechanisms above is given in Figure 7. The functional consequences of this interaction shift remain unknown and warrant ongoing studies.

## Conclusion & future perspective

Therapy resistance of Her2 amplified breast cancer against Her2 targeted therapies is becoming a relevant issue. Thus, the identification of resistance inducing signaling pathways and alternative therapeutic targets is of the utmost need. This study identifies the NFκB signaling as a possible target for future therapeutics. The crosstalk of NFκB signaling with other relevant signaling pathways, for example, MAPK signaling and Her2 signaling, still need to be identified and precisely described. This should be the subject of future studies.

**Summary points**

- NFκB inhibition on the IKK level using 5 μM IKK-16 severely affects the cell viability of SKBR3 cells but does not affect HaCat cells.
- PTPIP51 crosslinks the NFκB signaling to the MAPK pathway in SKBR3 cells.
- NFκB inhibition on IκB ubiquitin ligase level is bypassed by translocation of PTPIP51 into the MAPK pathway in SKBR3 cells.

## References

[1] Buchholz TA, Garg AK, Chakravarti N The nuclear transcription factor kappaB/bcl-2 pathway correlates with pathologic complete response to doxorubicin-based neoadjuvant chemotherapy in human breast cancer. Clin. Cancer Res. 11(23), 8398–8402 (2005). 16322301 10.1158/1078-0432.CCR-05-0885

[2] Mann AP, Verma A, Sethi G Overexpression of tissue transglutaminase leads to constitutive activation of nuclear factor-kappaB in cancer cells: delineation of a novel pathway. Cancer Res. 66(17), 8788–8795 (2006). 16951195 10.1158/0008-5472.CAN-06-1457

[3] Wang DJ, Ratnam NM, Byrd JC, Guttridge DC NF-κB functions in tumor initiation by suppressing the surveillance of both innate and adaptive immune cells. Cell Rep. 9(1), 90–103 (2014). 25263557 10.1016/j.celrep.2014.08.049 PMC4882153

[4] Pires BRB, Mencalha AL, Ferreira GM NF-kappaB is involved in the regulation of EMT genes in breast cancer cells. PLoS ONE 12(1), e0169622 (2017). 28107418 10.1371/journal.pone.0169622 PMC5249109

[5] Gilmore TD, Kalaitzidis D, Liang M-C, Starczynowski DT The c-Rel transcription factor and B-cell proliferation: a deal with the devil. Oncogene 23(13), 2275–2286 (2004). 14755244 10.1038/sj.onc.1207410

[6] Terzić J, Grivennikov S, Karin E, Karin M Inflammation and colon cancer. Gastroenterology 138(6), 2101–2114.e5 (2010). 20420949 10.1053/j.gastro.2010.01.058

[7] Xia Y, Shen S, Verma IM NF-κB, an active player in human cancers. Cancer Immunol. Res. 2(9), 823–830 (2014). 25187272 10.1158/2326-6066.CIR-14-0112 PMC4155602

[8] Merkhofer EC, Cogswell P, Baldwin AS Her2 activates NF-kappaB and induces invasion through the canonical pathway involving IKKalpha. Oncogene 29(8), 1238–1248 (2010). 19946332 10.1038/onc.2009.410 PMC2829103

[9] Moasser MM The oncogene *HER2*: its signaling and transforming functions and its role in human cancer pathogenesis. Oncogene 26(45), 6469–6487 (2007). 17471238 10.1038/sj.onc.1210477 PMC3021475

[10] Slamon DJ, Clark GM, Wong SG, Levin WJ, Ullrich A, McGuire WL Human breast cancer: correlation of relapse and survival with amplification of the *HER-2/neu* oncogene. Science 235(4785), 177–182 (1987). 3798106 10.1126/science.3798106

[11] Li Q, Verma IM NF-kappaB regulation in the immune system. Nat. Rev. Immunol. 2(10), 725–734 (2002). 12360211 10.1038/nri910

[12] Liu F, Xia Y, Parker AS, Verma IM IKK biology. Immunol. Rev. 246(1), 239–253 (2012). 22435559 10.1111/j.1600-065X.2012.01107.x PMC3311052

[13] Brobeil A, Kämmerer F, Tag C, Steger K, Gattenlöhner S, Wimmer M PTPIP51 – a new RelA-tionship with the NFκB signaling pathway. Biomolecules 5(2), 485–504 (2015). 25893721 10.3390/biom5020485 PMC4496682

[14] Dietel E, Brobeil A, Tag C, Gattenloehner S, Wimmer M Effectiveness of EGFR/HER2-targeted drugs is influenced by the downstream interaction shifts of PTPIP51 in HER2-amplified breast cancer cells. Oncogenesis 7(8), 64 (2018). 30139932 10.1038/s41389-018-0075-1 PMC6107558

[15] Brobeil A, Bobrich M, Tag C, Wimmer M PTPIP51 in protein interactions: regulation and *in situ* interacting partners. Cell Biochem. Biophys. 63(3), 211–222 (2012). 22544307 10.1007/s12013-012-9357-y

[16] Yu C, Han W, Shi T PTPIP51, a novel 14-3-3 binding protein, regulates cell morphology and motility via Raf-ERK pathway. Cell. Signal. 20(12), 2208–2220 (2008). 18771726 10.1016/j.cellsig.2008.07.020

[17] Brobeil A, Koch P, Eiber M, Tag C, Wimmer M The known interactome of PTPIP51 in HaCaT cells – inhibition of kinases and receptors. Int. J. Biochem. Cell Biol. 46, 19–31 (2013). 24501773

[18] Brobeil A, Bobrich M, Wimmer M Protein tyrosine phosphatase interacting protein 51–a jack-of-all-trades protein. Cell Tissue Res. 344(2), 189–205 (2011). 21369858 10.1007/s00441-011-1146-1

[19] Hayakawa M, Miyashita H, Sakamoto I Evidence that reactive oxygen species do not mediate NF-kappaB activation. EMBO J. 22(13), 3356–3366 (2003). 12839997 10.1093/emboj/cdg332 PMC165656

[20] Gupta SC, Sundaram C, Reuter S, Aggarwal BB Inhibiting NF-κB activation by small molecules as a therapeutic strategy. Biochim. Biophys. Acta 1799(10–12), 775–787 (2010). 20493977 10.1016/j.bbagrm.2010.05.004 PMC2955987

[21] Waelchli R, Bollbuck B, Bruns C Design and preparation of 2-benzamido-pyrimidines as inhibitors of IKK. Bioorg. Med. Chem. Lett. 16(1), 108–112 (2006). 16236504 10.1016/j.bmcl.2005.09.035

[22] Leuchowius K-J, Jarvius M, Wickström M High content screening for inhibitors of protein interactions and post-translational modifications in primary cells by proximity ligation. Mol. Cell. Proteomics 9(1), 178–183 (2010). 19864249 10.1074/mcp.M900331-MCP200 PMC2808263

[23] Xue Y, Ren J, Gao X, Jin C, Wen L, Yao X GPS 2.0, a tool to predict kinase-specific phosphorylation sites in hierarchy. Mol. Cell. Proteomics 7(9), 1598–1608 (2008). 18463090 10.1074/mcp.M700574-MCP200 PMC2528073

[24] Brobeil A, Chehab R, Dietel E, Gattenlöhner S, Wimmer M Altered protein interactions of the endogenous interactome of PTPIP51 towards MAPK signaling. Biomolecules 7(3), 55 (2017). 10.3390/biom7030055 PMC5618236 28754031

